# Morphinism

**Published:** 1900-03

**Authors:** C. C. Stockard

**Affiliations:** Atlanta, Ga.


ATLANTA

### Journal-Record of Medicine.

Successor to Atlanta Medical and Surgical Journal, Established 1855, and Southern Medical Record, Established 1870.

Vol. I. MARCH, 1900. No. 12.

EDITORS { BERNARD WOLFF, M.D., DUNBAR ROY, M.D. M. B. HUTCHINS, M.D., BUSINESS MANAGER.

PUBLISHED MONTHLY. OFFICE 305 AND 306 FITTEN BUILDING.

### *ORIGINAL COMMUNICATIONS.*

### MORPHINISM.*

By C. C. STOCKARD, M.D.,

Atlanta, Ga.

Of all the various drug addictions, the morphia is the most common, the most serious in its consequences, the most difficult to leave off, and the most likely when it is left off to be followed by relapse. As to whether it is more common than the whiskey habit I am not sure, but, in the above statement, reference was had to those substances generally considered drugs, while whiskey is usually looked upon as a beverage. I am by no means certain, however, that there are not more morphia habitués than there are of those who use whiskey to excess, that is, to the point of intoxication. Dr. Crothers, who has studied the question of drug addictions closely for a long time, says that one out of six of the physicians

*Read before the Atlanta Society of Medicine.

of the United States are habitual users of morphine. The proportion is much larger among physicians than among any other class, but still the number of people addicted to the drug is very large, and I fear it is still on the increase, though some good authorities, among them Dr. Mattison, think it is on the decline.

When we come to consider the causes of the great increase of this evil during the past three or four decades, there are several factors entering into it. The most potent has undoubtedly been the hypodermic syringe, which, by its almost magic-like power of relieving pain, has led to its use much too frequently. Insomnia, resulting from nervous debility, induced by the strain consequent upon up-to-date business methods and the struggle for money, is a very common excuse for getting into the habit, and, when begun for this cause, the probability of ever getting out of its thraldom is extremely small. This same business and social condition, the struggle to meet the extravagant methods of the day, leads frequently to other troubles—neurasthenia, neuralgia, indigestion, hemorrhoids, etc.—all of which are frequent causes of the establishment of morphia habituation.

The habitual use of morphia causes more serious consequences than any other drug so used, though the evidence of damage done the system, as shown by *post-mortem* examination, is very slight, the damage being chiefly one of function. The action of every part and organ of the body is interfered with, and the individual is unfitted for the duties of life, no matter what his occupation may be. He is entirely untrustworthy, for the reason that he will neglect anything for his accustomed dose. I do not believe that, as a rule, the habitué will lie, except on the one point of his habit; but when he is out of his drug, if necessary to obtain it, he will not hesitate to lie, steal, or even commit murder, to get it. When supplied with it, though, he is a harmless citzen even if he is also a useless one. His incapacity for the duties of life is mainly due to the fact that he can keep his mind for any length of time on only one subject, and that is morphia.

The habitual use of morphia gives rise to transient albuminuria, generally, but not always, to constipation, sometimes alternating with diarrhea or dysentery, emaciation, etc. It does not produce the marked structural changes that alcohol does, very slight evidences being discernible in any of the tissues on *post-mortem;* but still it shortens life materially by lowering vitality and resistive power, thus rendering the subject an easy victim to acute diseases, though it does seem almost to give immunity from some.

In making the statement that morphia is productive of more serious consequences than is the habitual use of any other drug, I am not unmindful that cocain damages far more rapidly; but in this case the effects so soon become apparent, and it so utterly unfits the subject for business, and even makes him dangerous to those around, that something must be done before the damage becomes irreparable; and the difficulties of leaving off cocain are not nearly so great as in the case of morphia, nor is relapse nearly so likely to occur.

As to the difficulty of leaving off morphia when one has become thoroughly addicted to its use very few have any real conception. We all know that nervousness, vomiting and diarrhea occur, but what these mean to the exalted sensitiveness of one whose nerves are reacting from the benumbed condition in which they have long been held, seems to be more than can be generally appreciated. Then there are actual pains in the back and legs. They all say they suffer the tortures of the damned. In fact, during the whole course of a gradual reduction, and for weeks after the end has been reached, the patient is a pitiable neurasthenic. When the drug is suddenly withdrawn the reaction is more prompt and rapid, so that one is about in the same condition one week after that one is a month after leaving off the drug by the gradual method. It is claimed that by the administration of bromides in large doses—ʒ ij every two hours for a few days—the worst of the sudden-withdrawal method is passed without suffering. I have never tried this plan, but by the use of strychnia and mydriatics, pushed to a sufficient degree, after having thoroughly cleared the alimentary canal, this method is practically robbed of its terrors, and, in a few days the patient has a good appetite, sleeps naturally, and builds up very much as one having had typhoid fever.

The most important thing to be considered in the treatment of these cases is the prevention of relapse. The majority can be carried through the leaving-off process by any of the methods in use, but the percentage of relapses is very large. When a patient returns home in a few weeks, still in a condition of profound neurasthenia, weak and nervous, with poor digestion and unable to sleep without hypnotics, and in this condition undertakes to do his usual work, he soon becomes discouraged and returns to his old habit. That these unfortunates are in earnest about wanting to get well is proven by their repeated efforts, and the money they spend in these efforts. One who came to me said he had taken eleven treatments and had spent six thousand dollars in his efforts to get well. He told me I could have all the time I wanted on his case, and he is now, more than eighteen months after, a well man, weighing when he came to me one hundred and fifteen pounds, and now one hundred and seventy.

The German "Nach Kur"—after-cure—is excellent. After having gone through the weaning-off process the patient is sent to one of these, where, free from the cares of business, he finds fresh air, pleasant surroundings, and agreeable diversions. If all could spend from three to six months in such a place the number of relapses would be greatly diminished. Unfortunately, however, most of these cases have, as a result of their habit, neglected business, run through with what they had and are not able to take such a course.

Another matter of first importance in the prevention of relapse is the removal, by a surgical operation, or other treatment, of any existing disease which will cause pain or suffering, as these will certainly lead to relapse, and if one is suffering from some trouble which cannot be relieved it is utterly useless to stop the drug.

Whereas I prefer in the majority of cases to stop the drug abruptly, there are cases in which a different course is better, and in these cases I try by every rational means, baths, electricity, massage and tonics, to build up the general health while the drug is being reduced. Whenever, though, the general condition is fairly good I prefer to stop the drug at once, and, as I said before, the reaction is more prompt and one builds up much more rapidly, while the worrying, teasing process of reduction is much harder to bear by the patient, and gives the attendants ten times the trouble. In conclusion I will give brief histories of a few cases treated during the past two years:

J. T. B., aet. 36, a robust, healthy looking man, a merchant who had become addicted to the use of morphia while using it for the relief of facial neuralgia, came to me August 1st, 1898. He also had hemorrhoids, which I advised him to have removed, but he said he would attend to it later as he must get through and return to his business as quickly as possible. I had not, at that time, begun using the sudden withdrawal method, so I began cutting him down, at the same time using eliminative and tonic treatment, but in about ten days he developed a malarial fever which lasted two weeks and during this time my reduction had to stop. After recovery from the fever I proceeded and had him off his drug in about six weeks from the time of arrival, and, much against my advice he returned home a week later, still very weak and in no condition to attend to business. In about three months he returned, saying that a month after leaving me he had an attack of tonsillitis, attended with severe facial neuralgia, for which his physician, who knew nothing of his habit, gave hypodermics of morphia, and of course he kept it up. This time I took him off at once, and in a week he went home, again earlier than I wanted him to. Not long after this he began suffering with rectal trouble, had abscesses, resulting in fistula which caused him great suffering for several months, during which time he was operated on for fistula and hemorrhoids, and, of course, resumed his habit. He returned to me again December 27th, 1899, saying he was now rid of his other troubles and thought he could keep well, if free from his drug habit. The night before leaving home, however, he had been up the whole night on account of a fire in his town, and the very day he came to me he developed a pneumonia in both lungs, which carried him off in a few days. This man, had he taken sufficient time at first to get rid of his hemorrhoids, and catarrh which caused his neuralgia, could have made a good recovery. He was not of the neurotic temperament which is necessary for the most intractable cases, he had a good constitution, and outside of the two troubles mentioned there was nothing that should or would likely have caused a relapse in his case.

A. F., aet. about 48, came to me July, 1898. Had used morphia five years, taking 15 grs. by the syringe daily. His arms and legs were full of scars, the result of abscesses. He was emaciated, weak, suffered from insomnia, for which he had been taking bromidia, and was altogether in a pitiable condition. He had been treated eleven times, and said he did not intend to make but one more trial. I took six weeks to get him off, at the same time giving him vapor baths, and such tonic and alterative treatment as seemed indicated. At the last of the reduction he was very weak, the pulse being hardly perceptible, but after a while reaction set in and he built up very satisfactorily and is now, more than eighteen months after, strong and well in every way, though he had a hard fight for a long time, and once fell, but he came to me when he had only taken two doses, and thus a relapse was prevented.

The cases below were all treated by the immediate withdrawal method.

H. F. B., aet. about 40, a robust healthy looking man, but of bilious temperament, had gotten into the morphia habit from its being given for the relief of gall-stone colic. Had been taking it six months, and was taking ten grains a day by the mouth. The drug was stopped at once, and in three or four days he had a good appetite and built up rapidly, leaving for home in two weeks. The last time heard from, he said his health was perfect and felt there was no danger of relapsing.

Mrs. W., a morphia-cocainist, had suffered for years from cystitis, for the relief of which the buttonhole operation had been done, and the cocain habit was established from applications to relieve the pain of urinating. She was emaciated, very nervous, mind considerably affected and altogether a most unpromising case. Both drugs were stopped at once. She had some hallucinations at night for a few days, and owing to a lot of business complications worrying her greatly, she did not, at first, build up as rapidly as she would have done. In a few weeks the improvement became more rapid, and in two and a half months from the beginning of the treatment she had gained about sixty pounds, and is now, nine months after treatment, well.

Dr. G., aet. about 30, a morphia-cocainist, had gotten into the habit simply by reason of taking something to brace him up for his work. This was not a very promising case, but his improvement was more prompt to set in and more rapid than any patient I have ever treated, gaining rapidly in weight, having all the time an enormous appetite, and never any diarrhea or digestive trouble. I have seen him recently, eight months after treatment, and he seems like a different man, perfectly well.

Dr. K. had become addicted to the use of morphia seven or eight years ago, and while in New York taking a post-graduate course went into a hospital and was treated. He remained well for about five years, when he developed the habit again, taking it on account of a very painful throat trouble. The throat affection having been relieved, he came to me a year afterward for relief from his habit. As showing the difference between the gradual reduction and the immediate withdrawal methods, I quote from a recent letter from him: "Your treatment is far beyond my expectations. No one knows so well as one that has tried other treatments and then tried yours." He was incredulous of being able to stop the drug at once, without great suffering, and came near leaving when he found that I proposed to treat him that way. However in two or three days reaction set in and he built up rapidly, going home in two weeks from the time of arrival, having gained some in weight, eating and sleeping well.

C. G. W., aet. about 35, had taken morphia and cocain for five years, and was taking forty grains of morphia per day by the mouth. Although taking this large amount, as he was in pretty good general condition, I did not hesitate to stop all his drugs at once. I find it easier, usually, in the cases that use morphia and cocain than in the simple morphinist, for the reason that when the cocain is stopped, a deep and prolonged sleep follows, instead of wakefulness, as in simple morphine cases. In this case, as seems to be usual too in the morphia-cocainist, there was almost entire absence of the digestive disturbances, vomiting and diarrhea, attendant upon morphia withdrawal. He slept most of the first three days and from that time on built up rapidly.

